# A Piezoelectric-Actuated Variable Stiffness Miniature Rotary Joint

**DOI:** 10.3390/ma18143289

**Published:** 2025-07-11

**Authors:** Yifan Lu, Yifei Yang, Xiangyu Ma, Ce Chen, Tong Qin, Honghao Yue, Siqi Ma

**Affiliations:** 1 State Key Laboratory of Robotics and System, Harbin Institute of Technology, Harbin 150001, China; yf.lu@hit.edu.cn (Y.L.); iffyyoung@stu.hit.edu.cn (Y.Y.); 23s108346@stu.hit.edu.cn (X.M.); block@hit.edu.cn (H.Y.); 2 Construction Machinery Manufacturing Co., Ltd., Guangxi Construction Engineering Group, Nanning 530299, China

**Keywords:** piezoelectric, variable stiffness, rotary joint, miniature, actuation

## Abstract

With the acceleration of industrialization, deformable mechanisms that can adapt to complex environments have gained widespread applications. Joints serve as carriers for transmitting forces and motions between components, and their stiffness significantly influences the static and dynamic characteristics of deformable mechanisms. A variable stiffness joint is crucial for ensuring the safety and reliability of the system, as well as for enhancing environmental adaptability. However, existing variable stiffness joints fail to meet the requirements for miniaturization, lightweight construction, and fast response. This paper proposes a piezoelectric-actuated variable stiffness miniature rotary joint featuring a compact structure, monitorable loading state, and rapid response. Given that the piezoelectric stack expands and contracts when energized, this paper proposes a transmission principle for stiffness adjustment by varying the pressure and friction between active and passive components. This joint utilizes a flexible hinge mechanism for displacement amplification and incorporates a torque sensor based on strain monitoring. A static model is developed based on piezoelectric equations and displacement amplification characteristics, and simulations confirm the feasibility of the stiffness adjustment scheme. The mechanical characteristics of various flexible hinge structures are analyzed, and the effects of piezoelectric actuation capability and external load on stiffness adjustment are examined. The experimental results demonstrate that the joint can adjust stiffness, and the sensor is calibrated using the least squares algorithm to monitor the stress state of the joint in real time.

## 1. Introduction

With the increasing diversification of human production and livelihood, deformable mechanisms have become increasingly vital. Common deformable mechanisms include multi-joint robotic arms [1,2,3], deployable space structures [4,5,6], and folding-wing aircraft [7]. These mechanisms can change their form to enhance environmental adaptability and achieve better performance. The actuation and transmission of deformable mechanisms rely on one or more joints, which serve as carriers for transmitting force or torque and the structural foundation for folding and unfolding, locking, and unlocking motions. The stiffness of the joint has a significant impact on the static and dynamic performance of the mechanism. Higher joint stiffness enables the mechanism to maintain better shape stability and reduce error accumulation, but it also increases movement resistance and energy consumption, as well as intensifying component wear. Conversely, lower stiffness makes the joint adapt to changes in the external environment, reducing damage caused by impacts or vibrations, while it may lead to greater vibrations and lower motion precision. Therefore, by adjustment joint stiffness flexibly [8,9,10,11], deformable mechanisms can exhibit compliant characteristics [12], enhancing their adaptability and robustness in complex and variable environments. This improves operational precision, reduces energy consumption, and ensures safety in human–machine interactions [13].

The principles of variable stiffness transmission can be classified into five major categories, including elastic force modulation type, non-contact force regulation type, pneumatic and hydraulic actuation type, interface mechanics effect type, smart material response type, etc. Elastic force modulation [14,15,16,17,18,19] adjusts joint stiffness by altering the effective lever arm or pre-tightening force of elastic elements within rigid transmissions. Non-contact force regulation [20,21,22] exploits magnetic forces, modulated via inter-magnet distance or coil current, to achieve rapid stiffness control. Pneumatic and hydraulic actuation [23,24,25,26,27] varies stiffness through air pressure adjustment in deformable chambers or precise hydraulic fluid flow regulation. Interface mechanics effects [28,29,30,31,32,33] manipulate apparent system stiffness by controlling interfacial interactions (e.g., friction, particle interlocking, adhesion forces) between materials. Smart material [24,25,26,27,28,29,30,31,32,33,34,35,36,37,38,39] responses leverage phase/structure transitions in stimuli-responsive materials (e.g., SMAs, ER/MR fluids) under external fields to enable dynamic stiffness tuning. In recent years, piezoelectric materials [40,41,42,43], due to their compact structure, rapid response, high driving force, and low sensitivity to environmental factors, have been widely used in various smart actuators, while they are rarely applied in variable stiffness joints. There still exist several technical challenges that need to be addressed in current research on variable stiffness joints. The mechanical structure of elastic force modulation mechanisms is complex, which makes it difficult to achieve miniaturization. Electromagnetic actuated joints are susceptible to interference from surrounding electromagnetic fields, leading to control instability. Pneumatic and hydraulic systems require large components, such as cylinders, and their adequate sealing remains problematic. The fabrication of specialized interfaces is challenging, and the reliability of force transmission is often poor. Shape memory materials suffer from poor heat dissipation, making it difficult to apply them for repeated actuation. Rheological materials, which require electric or magnetic field environments, tend to have large dimensions, and the sealing is difficult to ensure. Therefore, variable stiffness joints based on existing technologies are constrained by technical bottlenecks such as miniaturization, repeatability, response speed, and stability under environmental disturbances. These limitations hinder the application of variable stiffness joints in novel lightweight multi-joint deformable mechanisms.

To address the above issues, given the advantages of piezoelectric materials, such as fast response and large output force, this paper utilizes the converse piezoelectric effect. A novel method is proposed to achieve variable stiffness transmission by adjustment the squeezing and friction effects between active and passive components through the expansion and contraction of a piezoelectric stack driven by voltage. A piezoelectric-actuated variable stiffness miniature rotary joint (PVS-MiniRJ) is designed, featuring a compact structure and light weight, which provides a feasible scheme for the development of lightweight multi-joint deformable mechanisms. The joint integrates a strain sensor and a variable stiffness actuator internally, realizing the integration of load-bearing, driving, and sensing functions. Experiments demonstrate that the joint’s stiffness can be actively and rapidly adjusted, with the capability for multiple repeated actuations. The PVS-MiniRJ technology holds significant promise for applications requiring compact, intelligent articulation systems, particularly in lightweight deployable structures and multi-joint miniature manipulators for confined-space operations.

In Section 2, the stiffness adjustment scheme is introduced, and the structural composition of the joint is elucidated. In Section 3, the feasibility of the stiffness adjustment scheme is validated by mechanical modeling and simulation, and the influence of multiple variables on the stiffness characteristics is investigated. In Section 4, the joint testing apparatus is constructed, the variable stiffness capabilities of the joint are tested, and the monitoring of the load-bearing state is described. In Section 5, the conclusions are provided.

## 2. Stiffness Adjustment Scheme and Structure

This section systematically introduces the specific implementation of its stiffness adjustment scheme and elucidates the compositional details of its mechanical structure, thereby providing a foundational framework for subsequent analysis.

### 2.1. Stiffness Adjustment Scheme

**Figure 1:**
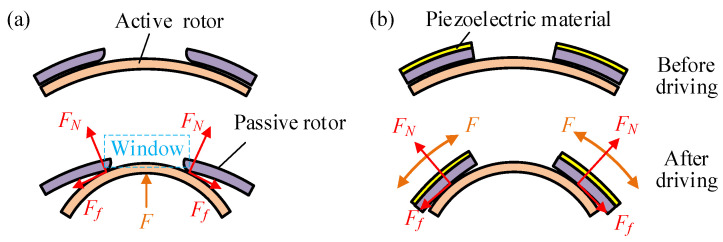
Two schemes of variable stiffness driving principles. (**a**) Expansion driving scheme actuated by piezoelectric stacks. (**b**) Bending driving scheme actuated by piezoelectric chips.

Based on the inverse piezoelectric effect, piezoelectric materials can generate force and displacement when an electric field is applied along the polarization direction of the dielectric. By varying the electric field, the interaction between the active and passive components can be altered, thereby modulating the transmission stiffness as the output characteristics change. Two force application schemes for piezoelectric material output are proposed, namely an expansion driving scheme and a bending driving scheme, as illustrated in Figure 1. These two schemes can be implemented through piezoelectric stacks and piezoelectric chips, respectively. The active and passive rotors are connected to the respective ends of the joint, transmitting torque through their interaction force. Expansion driving scheme utilizes an internally embedded piezoelectric actuator to generate radial force *F*, compressing the active rotor. This induces interfacial normal force *F_N_* and friction force *F_f_* at the active–passive rotor contact edges. External torque *T* further engages the rotors for torque transmission, and stiffness modulation occurs via piezoelectric force *F* controlling *F_N_* and *F_f_*. Bending driving scheme mounts piezoelectric elements on the passive rotor surface, producing a circumferential force *F* that bends the passive rotor. This deformation compresses the internal active rotor, regulating interfacial friction and compressive forces. Both schemes fundamentally adjust joint stiffness by tuning the piezoelectric output *F* to control rotor interaction forces. The expansion driving scheme imposes fewer structural requirements on the piezoelectric actuator, and the force transmission process is direct, which makes it easy to analyze. In contrast, the bending driving scheme involves a complex actuator structure, making the characteristic analysis more difficult. Therefore, the expansion driving scheme is adopted for stiffness adjustment in this study.

### 2.2. Driving and Transmission Path

**Figure 2:**
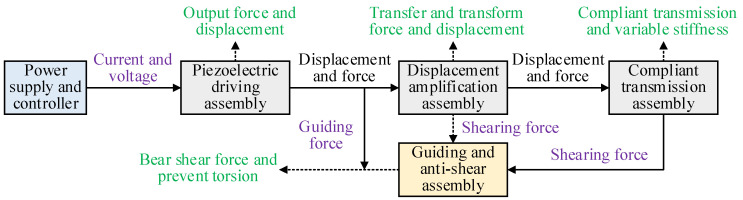
Transmission path and steps of electrical signals, forces, and displacements inside and outside the PVS-MiniRJ.

The main components of the PVS-MiniRJ include the piezoelectric driving assembly, displacement amplification assembly, compliant transmission assembly, guiding and anti-shear assembly, and state monitoring assembly. The transmission pathways for electrical signals, force, and displacement inside and outside the joint are depicted in Figure 2. The piezoelectric driving assembly, consisting of a piezoelectric stack and the control unit, outputs and adjusts force and displacement by controlling the voltage. The displacement amplification assembly, composed of several displacement amplification mechanisms, adjusts the magnitude and direction of the force and displacement produced by the piezoelectric driving assembly. The compliant transmission assembly, which includes the active rotor and the passive rotor, is the functional component for flexible transmission and stiffness adjustment. The guiding and anti-shear assembly, made up of shear-resisting and guiding components, bears the shear forces and prevents large torsion within the joint structure. Strain sensors are used in the state monitoring assembly to monitor the real-time load conditions.

### 2.3. Structure-and-Assembly Relationship

**Figure 3:**
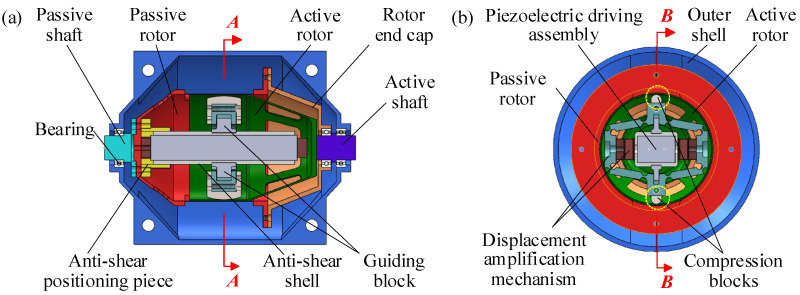
Overall sectional views of the PVS-MiniRJ. (**a**) *B*-*B* Axial sectional view. (**b**) *A*-*A* Radial sectional view.

Figure 3 illustrates the cross-sectional views of the overall assembly of the PVS-MiniRJ. The passive shaft is connected to the passive rotor, while the active shaft is connected to the active rotor. The rotor end cap, attached to the outer edge of the passive rotor, is supported by bearings mounted on the passive shaft. The entire joint is enclosed by an outer shell, which features a longitudinal split structure for installation, disassembly, and internal observation. External torque is input through the active shaft and transmitted to the active rotor. The torque passes through the piezoelectric driving assembly, displacement amplification assembly, and compliant transmission assembly, eventually being transferred to the passive shaft. As the output of the piezoelectric driving assembly varies, the interaction between the rotors changes accordingly, leading to an adjustment in stiffness. The relative positions of the passive rotor, anti-shear positioning pieces, and the first-stage amplification mechanism are fixed and connected in stages, ensuring a constant relative angle between the displacement amplification assembly and the passive rotor. The compression blocks rotate together with the passive rotor. The force and displacement output of the compression blocks align with the windows on the circular section of the passive rotor, maximizing the deformation capability of the active rotor.

#### 2.3.1. Piezoelectric Driving Assembly

Considering the performance in terms of size, output force, and displacement, the square MTP150/5×5/30 piezoelectric ceramic stack actuator produced by CoreMorrow Ltd. (Harbin, China) serves as the driving device. The technical parameters are listed in Table 1. This actuator is composed of a stack of 15 piezoelectric ceramic plates. Theoretical response time for a single actuation of the piezoelectric driving assembly, according to the resonance frequency, is in the microsecond range, indicating that the stiffness adjustment process is extremely fast. The input voltage at both ends of the piezoelectric stack is controlled by an E53.C piezoelectric controller (CoreMorrow Ltd., Harbin, China). The maximum output voltage of this controller is 150 V, and its average current is 60 mA.

#### 2.3.2. Displacement Amplification Assembly

**Figure 4:**
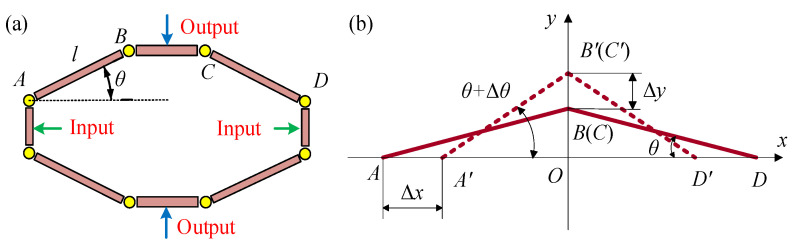
Triangular bridge amplification mechanism based on flexible hinges. (**a**) Displacement amplification structure composition. (**b**) Principle of displacement amplification.

The displacement output by the piezoelectric stack is at the micron level and has minimal impact on the deformation of the compliant transmission assembly. Therefore, a displacement amplification assembly is designed for magnifying the small displacement and transferring the force and displacement generated by the piezoelectric driving assembly to the compliant transmission assembly. The displacement amplification assembly utilizes a triangular bridge amplification mechanism [44,45,46,47,48] based on flexible hinges, and the structure and amplification principle are illustrated in Figure 4a,b. *AB*, *BC*, and *CD* can be approximately regarded as rigid rods connected by flexible hinges at points *A*, *B*, *C*, and *D*. The angle *θ* represents the angle between *AB* and *AD*, while Δ*θ* indicates the relative rotational angle of the rods during the movement. *x* and *y* represent the directions of power input and output, respectively.

From the geometric relationships in Figure 4, the theoretical amplification ratio *λ* of the bridge-type amplification structure could be derived as

(1) $$\lambda =\frac{\Delta y}{\Delta x}=\left|\frac{l\left[\sin\left(\theta +\Delta \theta \right)-\sin\theta \right]}{l\left[\cos\theta -\cos\left(\theta +\Delta \theta \right)\right]}\right|=\frac{1}{\tan\left(\theta +\frac{\Delta \theta }{2}\right)}$$

**Figure 5:**
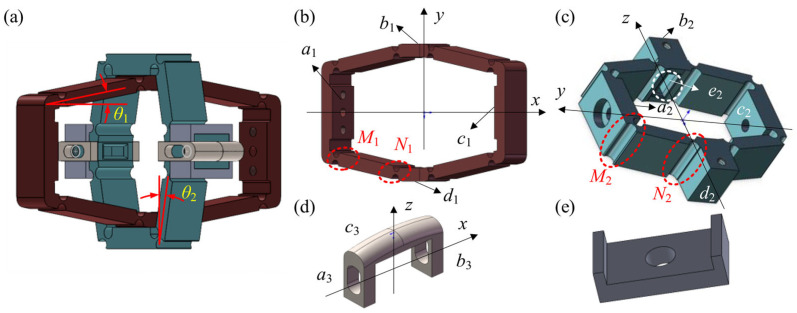
Structural characteristics and assembly relationship between components in the displacement amplification assembly. (**a**) Overall assembly. (**b**) First-stage displacement amplification mechanism. (**c**) Second-stage displacement amplification mechanism. (**d**) Compression block. (**e**) Limit block.

When Δ*θ* is very small, $\theta +\frac{\Delta \theta }{2}\approx \theta$. Then, the theoretical amplification ratio could be approximately expressed as $\lambda \approx \frac{1}{\tan\theta }=\cot\theta$. Therefore, the amplification ratio *λ* is mainly influenced by *θ*, which increases along with *θ*. A multi-stage displacement amplification mechanism can be constructed as the displacement amplification component to enhance the amplification effect. However, in practice, the flexible hinge cannot be simply regarded as a free rotational pair, and additional energy is required to overcome its deformation. If the amplification mechanism has too many stages, more energy from the piezoelectric stack will be lost in the amplification process. In this study, the displacement amplification component consists of a two-stage amplification mechanism containing compression blocks, limit blocks, and other structures, as shown in Figure 5. The total displacement amplification ratio of the mechanism could be written as

(2) $$\lambda_{\sum }=\lambda_{1}\lambda_{2}$$

where *λ*_1_ and *λ*_2_ represent the amplification ratios of the first-stage and second-stage displacement amplification mechanisms, respectively.

The two-stage displacement amplification mechanism converts piezoelectric axial outputs into radial actuation for rotors. In Figure 5a, *θ*_1_ = 8.3° and *θ*_2_ = 13°. For the first-stage displacement amplification mechanism in Figure 5b, *M*_1_ and *N*_1_ represent flexible hinges. In the *x*-direction, surface *a*_1_ and *c*_1_ are in contact with both ends of the piezoelectric stack, and these surfaces are regarded as the input end of the first-stage displacement amplification mechanism. In the *y*-direction, surface *b*_1_ and *d*_1_ are taken as the output end of the first-stage displacement amplification mechanism, which are connected to the input end of the second-stage displacement amplification mechanism. A cylindrical pinhole is at the center of surface *a*_1_ for the centering location. Through the first-stage displacement amplification mechanism, the force and displacement output by the piezoelectric stack are converted from the axial direction (*x*-direction) to the radial direction (*y*-direction). For the second-stage displacement amplification mechanism shown in Figure 5c, *M*_2_ and *N*_2_ represent flexible hinges. In the *y*-direction, surface *a*_2_ and *c*_2_ are connected to surface *b*_1_ and *d*_1_ of the first-stage amplification mechanism via bolts. In the *z*-direction, surface *b*_2_ and *d*_2_ are connected to the compression block. Thus, the force and displacement outputs are converted from the *y*-direction to the *z*-direction. The second-stage displacement amplification mechanism is engaged with the guiding and anti-shear assembly, and rectangular slots *e*_2_ are dug on surface *b*_2_ and *d*_2_ to prevent relative torsion between the piezoelectric driving assembly and the displacement amplification assembly. The side of the second-stage displacement amplification mechanism is bolted to the compression blocks.

In addition to the amplification mechanisms, auxiliary components such as compression blocks and limit blocks also play an indispensable role in the transmission process. The force and displacement output from the piezoelectric driving assembly are transmitted through the displacement amplification mechanism, applying pressure to the rotors of the compliant transmission assembly via the compression blocks shown in Figure 5d. The U-shaped holes on the feet *a*_3_ and *b*_3_ are securely bolted to the second-stage displacement amplification assembly. By adjustment the relative position between the compression block and the bolts, the initial gap between the arc-shaped surface *c*_3_ and the inner surface of the active rotor can be eliminated. In the *z*-direction, the compression blocks apply force to the compliant transmission assembly, causing the active rotor to deform and generate compression friction with the passive rotor, thus transmitting force and displacement between the active and passive shafts of the PVS-MiniRJ. The displacement and force output from the piezoelectric driving assembly are eventually transmitted to the compliant transmission assembly through the compression blocks. When the active shaft of the PVS-MiniRJ rotates relative to the passive shaft, the compression blocks are subjected to a frictional force. Then the displacement amplification assembly tends to twist around the *x*-axis, which makes the direction of force and displacement output unstable. Therefore, two pairs of limit blocks, shown in Figure 5e, are set up in order to restrict the degrees of freedom of the compression blocks, except for moving along the *z*-axis. There are two limit blocks in each pair, which are secured and tightly fitted to either side of the compression blocks using screws.

#### 2.3.3. Compliant Transmission Assembly

**Figure 6:**
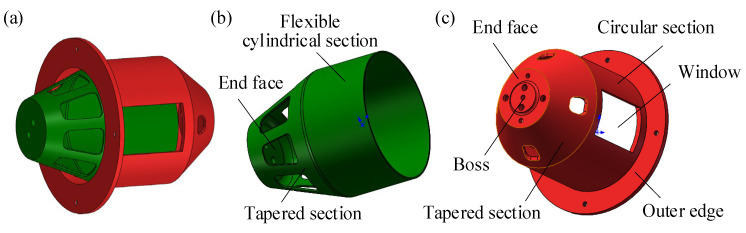
Structural characteristics and assembly relationship between components in compliant transmission assembly. (**a**) Assembly. (**b**) Active rotor. (**c**) Passive rotor.

The compliant transmission assembly mainly consists of an active rotor and a passive rotor, as in Figure 6a. The active rotor is composed of an end face, a tapered section, and a flexible cylindrical section, as shown in Figure 6b. The end face connected to the active shaft transmits external torque loads. Several grooves are arranged in a circular array on the tapered section. These grooves are used to increase flexibility, reduce the restriction on the radial deformation of the flexible cylindrical section, and reduce weight. The flexible cylindrical section is deformable with a thinner thickness. The inner surface comes into contact with the compression blocks and deforms under pressure. Meanwhile, the outer surface fits well with the passive rotor by adjustment dimensions and assembly tolerances. The passive rotor consists of an end face, a tapered section, a circular section, and an outer edge, as shown in Figure 6c. The circular section has two symmetrical windows, and the active rotor’s outer surface exerts the compressive and frictional force at the edges of these windows. Rounded corners are designed at the window edges to reduce stress concentration and prevent the internal active rotor from being damaged by sharp edges. The outer edge structure at the tail of the circular section is used to connect the rotor end cap. The inner side of the end face connects to the displacement amplification assembly, while the boss on the outer side connects to the passive shaft. The central cylindrical hole is used for positioning, and the hole in the tapered section is for wiring and power supply.

#### 2.3.4. Guiding and Anti-Shear Assembly

**Figure 7:**
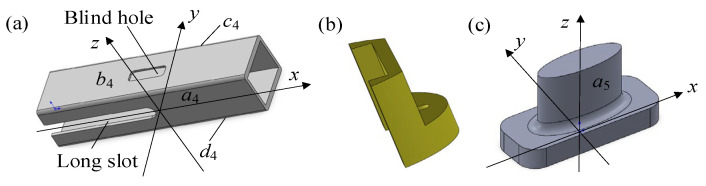
Structural characteristics and assembly relationship between components in guiding and anti-shear assembly. (**a**) Anti-shear shell. (**b**) Anti-shear positioning piece. (**c**) Guiding block.

The piezoelectric stack connected to the displacement amplification assembly cannot withstand significant shear forces. Since the displacement amplification assembly is in direct contact with the compliant transmission assembly, it is subjected to a torsional moment, which could potentially damage the piezoelectric stack. Therefore, the guiding and anti-shear assembly is used instead of the stack to bear the shear forces and provide positioning and guiding functions. The guiding and anti-shear assembly consists of an anti-shear shell, two anti-shear positioning pieces, and two guiding blocks. As shown in Figure 7a, the anti-shear shell is a hollow structure that encases and protects the piezoelectric driving assembly. Since the piezoelectric stack is conductive, except for its two ends, the anti-shear shell is fabricated from insulated reinforced polycarbonate material. To facilitate the power supply for the piezoelectric stack, long slots are carved on faces *a*_4_ and *c*_4_, while blind holes are located on faces *b*_4_ and *d*_4_. The anti-shear positioning pieces serve to fix the anti-shear shell and resist torque, as shown in Figure 7b. The bottom is connected to the passive shaft and the passive rotor, and the grooves are mounted on the outside of the anti-shear shell. The anti-shear positioning pieces bear the torsional moment together with the anti-shear shell. They are used for positioning, securing the anti-shear shell and protecting the piezoelectric stack. As illustrated in Figure 7c, the guiding block features an elliptical cylindrical surface *a*_5_, which fits with the rectangular slot *e*_2_ of the secondary amplification mechanism. Two guiding blocks guide the compression blocks installed at both ends of the secondary amplification mechanism to move along the *z*-direction. Line contacts between *a*_5_ and *e*_2_, instead of excessive surface contact, could prevent the motion pair from clamping stagnation. The bottom of the guiding block is fitted into the blind hole of the anti-shear shell and is firmly bonded with a strong adhesive.

#### 2.3.5. State Monitoring Assembly

**Figure 8:**
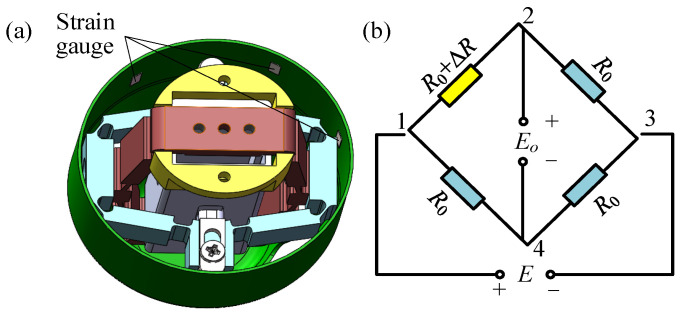
Strain gauge layout and circuit diagram. (**a**) Strain gauge position on the active rotor. (**b**) Strain gauge bridge circuit.

The stiffness of the PVS-MiniRJ is related to the torque and the relative angular displacement between the active and passive ends. For a position-controllable joint, real-time monitoring of the torque is crucial for controllable adjustment of stiffness. During operation, bulky torque sensors would consume considerable energy and encroach on payload mass and space. Hence, developing a miniaturized sensing method becomes necessary. In the process of achieving variable stiffness, the deformation of the active rotor is induced by the combined effects of the driving force from the piezoelectric assembly and the external torque load. Therefore, strain gauges are affixed to specific locations on the active rotor to reflect the joint torque by monitoring the strain values and the piezoelectric voltage. Possible locations for placing the strain gauges are illustrated in Figure 8a.

Since the deformation of the active rotor is related to the torque *T* and the driving force *F* of the piezoelectric stack, a functional relationship exists among the torque, the force, and the strain *ε* generated in the active rotor. This relationship can be expressed as

(3) ε=f(T,F)

The resistance of the strain gauge varies with the deformation of the active rotor. The relationship between strain and resistance is

(4) $$\frac{\Delta R}{R_{0}}=K_{0}\varepsilon$$

where Δ*R* represents the change in resistance of the strain gauge, *K*_0_ denotes the sensitivity coefficient of the metal wire of the strain gauge, and *R*_0_ is the initial resistance of the strain gauge. The strain gauge is connected to a Wheatstone bridge in a quarter-bridge configuration, as shown in Figure 8b. According to the principles of Wheatstone bridge measurement, the relationship between the real-time electrical resistance *R* of the strain gauge, the supply voltage *E_o_* of the bridge circuit, and the bridge measurement voltage *E* is

(5) $$E=\frac{\Delta R}{4R_{0}}E_{o}$$

The driving force *F* of the piezoelectric stack is related to the input voltage *U*. Combining Equations (3)–(5), the functional relationship between the input voltage *U* of the piezoelectric stack, the measurement voltage *E* of the strain gauge, and the torque *T* can be derived. Therefore, by monitoring *E* and *U*, the torque *T* is obtained for real-time monitoring of the load state of the PVS-MiniRJ.

## 3. Mechanical Modeling and Simulation Analysis

Building upon the stiffness adjustment scheme and structural composition detailed in Section 2, this section validates the feasibility of the proposed scheme through mechanical modeling and simulation, while investigating how multiple variables collectively influence the joint’s characteristics.

### 3.1. Mechanics Modeling of Adjustment Stiffness Process

**Figure 9:**
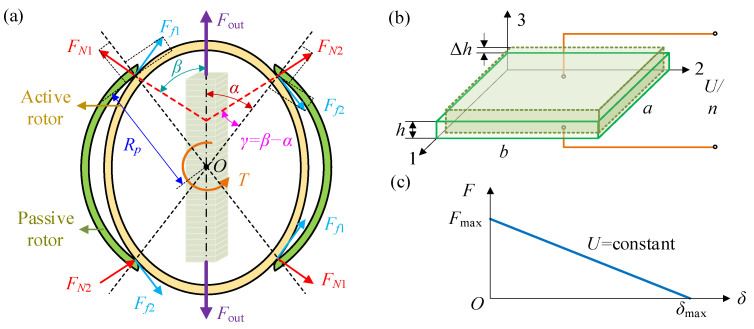
Static mechanical model of the PVS-MiniRJ, including force transfer relation. (**a**) The output of the piezoelectric stack actuator causes the interaction between the driving rotor and the driven rotor to change. (**b**) The change of the piezoelectric ceramic plate under the action of a unidirectional electric field. (**c**) The relation curves of driving force, deformation, and voltage output by piezoelectric ceramic.

When the PVS-MiniRJ is under static loading, the mechanical model can be expressed as shown in Figure 9a. Ideally, the loading state is centrally symmetric around the axis *O*. The output force *F*_out_ from the piezoelectric stack through the compression block leads to an interaction between the active rotor and the passive rotor. *F_N_*_1_ and *F_N_*_2_ represent the mutual compressive forces between the active rotor and the passive rotor. *F_f_*_1_ and *F_f_*_2_ represent the frictional forces. *T* is the torque acting on the active rotor. *α*, *β*, and *γ* are quantities representing the directions of the forces. *R_p_* is the distance between the contact point of the active rotor and the passive rotor and the axis *O*.

According to the force balance condition,

(6) $$\left(F_{N1}+F_{N2}\right)\cos\gamma \cos\alpha +\left(F_{f1}-F_{f2}\right)\sin\gamma \cos\alpha =F_{\mathrm{out}}$$

(7) $$\left(F_{N1}-F_{N2}\right)\cos\gamma =\left(F_{f1}+F_{f2}\right)\sin\gamma$$

Equations (6) and (7) indicate that variations in the output force *F*_out_ lead to changes in the compressive forces *F_N_*_1_ and *F_N_*_2_, as well as the frictional forces *F_f_*_1_ and *F_f_*_2_. These alterations subsequently modify the deformation state of the active rotor. Consequently, the interaction between the active rotor and the passive rotor changes, which causes the ability to adjust the joint stiffness. According to the principle of torque equilibrium,

(8) $$\left[2\left(F_{N2}-F_{N1}\right)\sin\gamma +2\left(F_{f1}+F_{f2}\right)\cos\gamma \right]R_{p}=T$$

Equation (8) shows that a change in the external torque will also affect the extrusion forces *F_N_*_1_ and *F_N_*_2_ and the friction forces *F_f_*_1_ and *F_f_*_2_.

From the above analysis, it can be seen that the output force *F*_out_ of the compression block is very important for the stiffness adjustment ability of the PVS-MiniRJ. Therefore, the force analysis of the piezoelectric driving process should be carried out. In the piezoelectric driving assembly, the piezoelectric stack is composed of several piezoelectric ceramic plates. As shown in Figure 9b, *a*, *b*, and *h* represent the length, width, and height of the piezoelectric ceramic plates, respectively, with the polarization direction along the height. According to the inverse piezoelectric effect, the stack contracts in directions 1 and 2, and extends in direction 3 when energized. The piezoelectric ceramic plates are influenced by both the stress field and the electric field, resulting in a strain that combines the elastic strain *ε*_1_ and the piezoelectric strain *ε*_2_. Therefore, the deformation of a single piezoelectric ceramic plate under the influence of the electric field is

(9) $$\delta =\varepsilon h=\left(\varepsilon_{1}+\varepsilon_{2}\right)h=\left(\frac{\sigma }{E_{0}}+d_{33}E_{3}\right)h$$

where *ε* is the strain along the 3-axis, *σ* is the stress along the 3-axis, *E*_0_ is the elastic modulus of the piezoelectric material, *d*_33_ is the piezoelectric strain coefficient, and *E*_3_ is the electric field strength along the 3-axis.

If the piezoelectric stack is composed of *n* layers of piezoelectric ceramic plates, the relationship between the voltage *U* applied across the ends of the stack and the electric field strength *E*_3_ along the 3-direction is given by

(10) $$U=nE_{3}h$$

Substituting Equation (10) into Equation (9), the expression for the stress *σ* along the 3-direction is given by

(11) $$\sigma =\frac{\delta E_{0}}{h}-\frac{d_{33}E_{0}U}{nh}=\frac{E_{0}}{h}\left(\delta -d_{33}\frac{U}{n}\right)$$

The area of the piezoelectric ceramic plate is *A*, then the output driving force *F* can be derived as

(12) $$F=-\sigma A=\frac{E_{0}A}{h}\left(d_{33}\frac{U}{n}-\delta \right)$$

The relationship between the driving force *F*, displacement *δ*, and voltage *U* for the piezoelectric ceramic, as derived from Equation (12), is shown in Figure 9c. For a piezoelectric stack composed of *n* layers of piezoelectric ceramic plates, by combining Equations (9) and (10), the displacement generated along the 3-axis under the excitation of the electric field can be derived as

(13) $$\delta_{\sum }=n\delta =n\varepsilon h=n\left(\frac{\sigma }{E_{0}}+d_{33}\frac{U}{h}\right)h$$

Based on Equation (12), the total driving force *F*_∑_ output by the piezoelectric stack equals *F*. According to the property of the displacement amplification mechanisms, *x*_out_ = *λ*_∑_*δ*_∑_. Therefore, the relationship between the voltage *U*, the output force *F*_out_, and the displacement *x*_out_ of the compression block is given by

(14) $$F_{\mathrm{out}}=\frac{F_{\Sigma }}{\lambda_{\Sigma }}=\frac{E_{0}Ad_{33}}{nh\lambda_{\Sigma }}U-\frac{E_{0}A}{nh{\lambda_{\Sigma }}^{2}}x_{\mathrm{out}}$$

Intuitively, the output driving force *F*_out_ and output displacement *x*_out_ by the compression block seem to be beneficial to the increase of joint stiffness. However, for a specific structure, *F*_out_ and *x*_out_ should have a deterministic relationship, namely *F*_out_ = *F*_out_ (*x*_out_) or *x*_out_ = *x*_out_ (*F*_out_). Therefore, Equation (14) can be transformed into *F*_out_ = *F*_out_ (*U*) or *x*_out_ = *x*_out_ (*U*). According to the principle of variable stiffness, the deformation of the active rotor affected by *F*_out_ or *x*_out_ is correlated with the rotational stiffness *K* of the PVS-MiniRJ. Thus, voltage *U* can be used as the control variable and the deformation of the active rotor as the measurement variable, and then the stiffness can be adjusted through voltage regulation.

### 3.2. Force Analysis of Flexible Hinge

**Figure 10:**
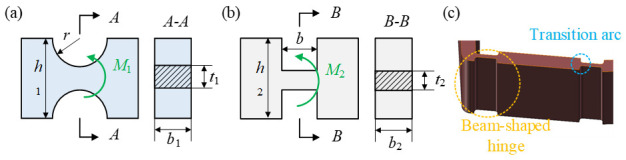
Schematic diagram of flexible hinges. (**a**) Circular-shaped flexible hinge. (**b**) Beam-shaped flexible hinge. (**c**) Structural details of the improved beam-shaped hinge through transition arcs.

In the displacement amplification assembly, the rotational stiffness of the flexible hinge is reduced by local notches so that it can play a similar role to a rotational pair. The shapes of flexible hinges include circular form, beam form, etc., as shown in Figure 10. The widths of the connected components are *h*_1_ and *h*_2_, the thicknesses or minimum thicknesses of the hinges are *t*_1_ and *t*_2_, the radius of the circular hinge is *r*, and the length of the beam hinge is *b*. Although the local weakening approach enhances the structural elastic deformation capability and achieves displacement amplification, it also results in decreased strength and stiffness, thereby increasing the likelihood of stress concentration and reducing the load-bearing capacity. In this study, the impact of different flexible hinge structural configurations on the stress conditions of the displacement amplification mechanism is examined by finite element simulation analysis. To alleviate the local stress concentration, a circular arc transition is applied between the beam-shaped hinge and the connected components, thereby replacing the structure shown in Figure 10b with that in Figure 10c.

**Figure 11:**
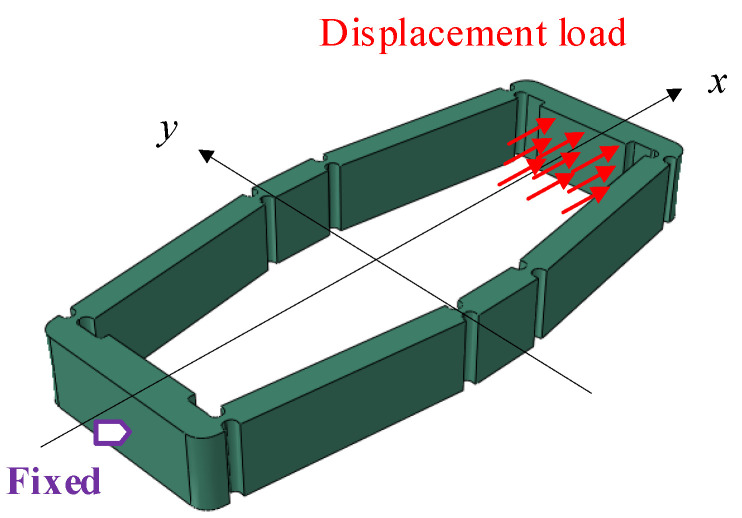
Load and constraint settings of the first-stage displacement amplification mechanism.

**Figure 12:**
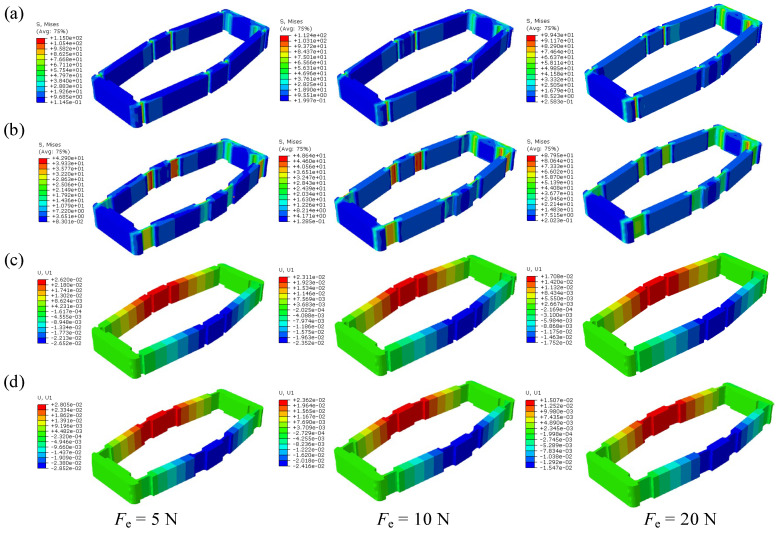
Static simulation results of the first-stage displacement amplification mechanism, including stress and displacement. (**a**) Stress distribution of the first-stage displacement amplification mechanism with circular-shaped hinges. (**b**) Stress distribution of the first-stage displacement amplification mechanism with beam-shaped hinges. (**c**) Displacement distribution of the first-stage displacement amplification mechanism with circular-shaped hinges along the *y*-direction. (**d**) Displacement distribution of the first-stage displacement amplification mechanism with beam-shaped hinges along the *y*-direction.

The displacement amplification mechanism is made of 304 stainless steel. A controlled variable method is employed to investigate the impact of flexible hinge shapes on the amplification effect and stress conditions. The simulation settings are shown in Figure 11. In the input direction of the first-stage displacement amplification mechanism (the *x*-direction in Figure 5b), one end is fixed while a forced displacement load of 0.01 mm is applied at the other end to simulate the displacement output of the piezoelectric stack. Meanwhile, in the output direction (the *y*-direction in Figure 5b), equal forces *F*_e_ are applied to both sides to simulate the obstructive effect of the rotors on the deformation of the displacement amplification mechanism. The simulation results of the first-stage amplification mechanism are shown in Figure 12.

In Table 2, the dimensional parameters, the maximum equivalent stress σVmax, and the calculated displacement amplification ratios *λ*_1_ are presented. It can be observed that stress concentration occurs around the flexible hinges due to the weakness of the structure at these points. The type of elastic hinge seems to have similar effects on the displacement amplification ratios. However, because the length dimension of the weak structure in the beam-shaped elastic hinge is larger than that in the circular-shaped hinge, the stiffness is reduced, resulting in increased deflection and a smoother displacement transition. As a result, the stress conditions for the beam-shaped hinge are better than those of the circular-shaped hinge. Consequently, the beam-shaped flexible hinge structure is employed in both the first-stage and second-stage amplification mechanisms.

### 3.3. Simulation Analysis

**Figure 13:**
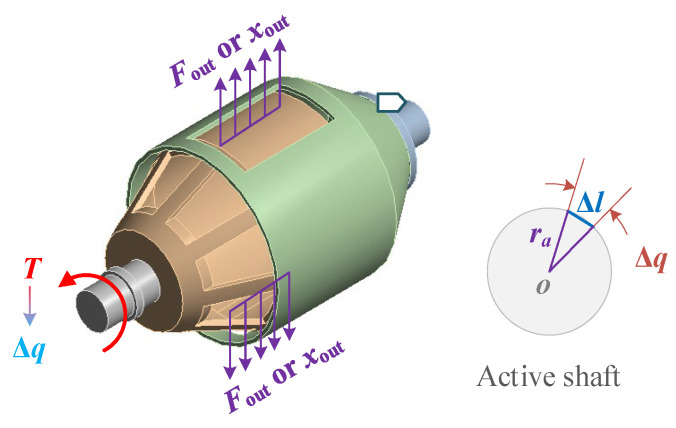
Load and constraint settings of the stiffness adjustment mechanism.

A simplified stiffness adjustment mechanism is formed by the active shaft, passive shaft, active rotor, and passive rotor. The mechanical characteristics of the stiffness adjustment mechanism are analyzed by using finite element simulation. The simulation setup is shown in Figure 13. The passive shaft is fixed, and torque *T* is applied at the active shaft end. Meanwhile, either radial driving force *F*_out_ or displacement *x*_out_ is applied to the active rotor, and the rotational behavior of the active shaft is measured. According to the maximum linear displacement Δ*l* of the active shaft along the axis direction and the value of the radius *r*_a_ of the driving shaft in the compliant transmission assembly, the relative rotation angle between the active shaft and the passive shaft is calculated as

(15) $$\Delta q=\frac{\Delta l}{r_{a}}$$

According to the definition of stiffness, the formula for calculating the rotational stiffness *K* of the PVS-MiniRJ equals the ratio of the transmitted torque *T* to the relative angular displacement Δ*q* between the active and passive shafts, which can be expressed as

(16) $$K=\frac{T}{\Delta q}$$

#### 3.3.1. Influence of *F*_out_ on Stiffness Adjustment

**Figure 14:**
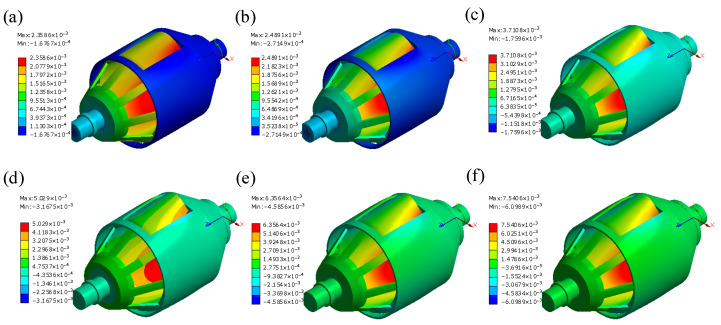
Circumferential displacement nephograms of the stiffness adjustment mechanism around the joint axis when a torque *T* = 10 N·mm is applied at the active shaft, and the radial driving force *F*_out_ applied to the active rotor is varied. (**a**) *F*_out_ = 3 N. (**b**) *F*_out_ = 5 N. (**c**) *F*_out_ = 10 N. (**d**) *F*_out_ = 15 N. (**e**) *F*_out_ = 20 N. (**f**) *F*_out_ = 25 N.

**Figure 15:**
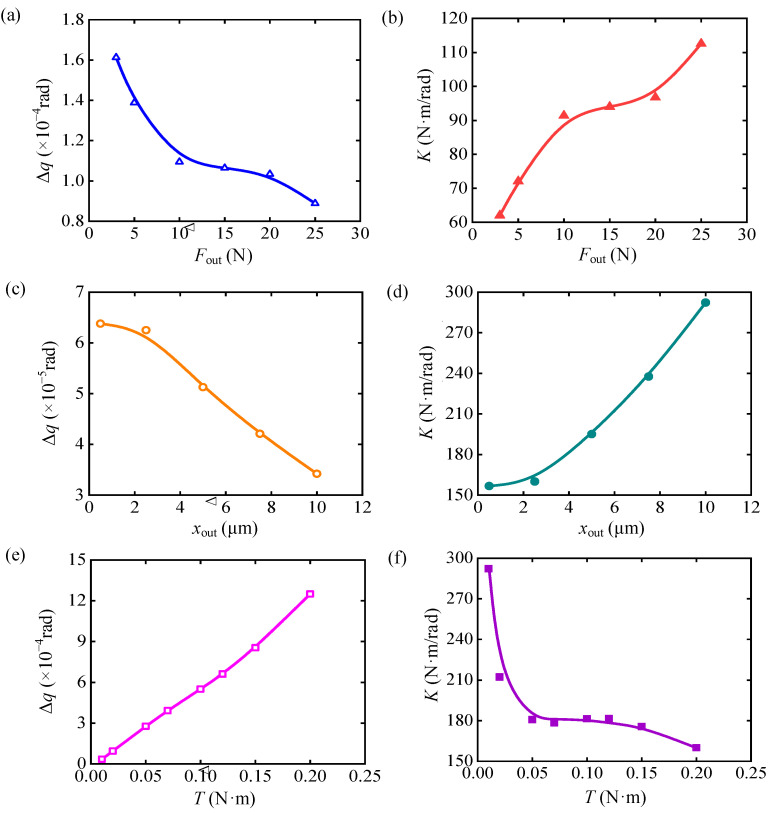
The influence of driving force *F*_out_, piezoelectric output displacement *x*_out_, and joint torque *T* on the flexible rotation angle Δ*q* and output stiffness *K* of the stiffness adjustment mechanism. (**a**) *F*_out_ − Δ*q* curve. (**b**) *F*_out_ − *K* curve. (**c**) *x*_out_ − Δ*q* curve. (**d**) *x*_out_ − *K* curve. (**e**) *T* − Δ*q* curve. (**f**) *T* − *K* curve.

When a torque *T* = 10 N·mm is applied at the active shaft and the radial driving force *F*_out_ applied to the active rotor is varied, the simulation produces the circumferential displacement cloud diagrams of the stiffness adjustment mechanism around the PVS-MiniRJ axis, as shown in Figure 14. It can be clearly observed that there is an interaction between the active rotor and the passive rotor, including squeezing at the window edge, and this contact force allows the active rotor to transmit torque or motion to the passive rotor. Based on the simulation results and Equation (15), the relationship between the rotation angle Δ*q* and the driving force *F*_out_ is obtained, as illustrated in Figure 15a. By using Equation (16) to calculate the joint stiffness *K*, the relationship between *K* and driving force *F*_out_ is shown in Figure 15b. The results indicate that, as the force output *F*_out_ from the piezoelectric stack increases during the operation of the piezoelectric driving assembly, the joint stiffness also increases. This is because the greater the force output, the stronger the extrusion effect between the active rotor and the passive rotor. As a consequence, the ability of the passive rotor to hinder the rotation of the active rotor becomes more prominent, resulting in increased macroscopic rotational stiffness of the joint.

#### 3.3.2. Influence of *x*_out_ on Stiffness Adjustment

When a torque *T* of 10 N·mm is applied at the active shaft end and the radial output displacement *x*_out_ is varied, the simulation results depict the relationships between the rotation angle Δ*q*, joint stiffness *K*, and piezoelectric output displacement *x*_out_, as shown in Figure 15c,d. It is evident that during the operation of the piezoelectric driving assembly, as the piezoelectric stack’s output displacement *x*_out_ increases within a certain operating range, the joint stiffness *K* also increases accordingly. Similarly, when the piezoelectric stack generates greater displacement output *x*_out_ through the compression block, the radial deformation of the active rotor increases. This enhances the compressive interaction between the active and passive rotors, thereby strengthening the passive rotor’s ability to resist the active rotor’s rotation. Consequently, the rotational stiffness of the joint is significantly increased.

#### 3.3.3. Influence of *T* on Stiffness Adjustment

With a piezoelectric stack output displacement of *x*_out_ = 0.01 mm, the torque load *T* on the active shaft is gradually increased, and the resulting variations in rotation angle Δ*q* and joint stiffness *K* are shown in Figure 15e,f. It can be observed that, with the output displacement remaining constant, the joint stiffness decreases as the external torque rises to an increasing degree. This phenomenon occurs because, as the external torque increases, frictional slip arises between the active and passive rotors, resulting in an increase in the relative rotation angle and a corresponding decrease in rotational stiffness.

### 3.4. The Purpose of Modeling and Simulation

Since the PVS-MiniRJ consists of various miniature non-standard components, couples extrusion and friction effects, performs significant contact nonlinearity, and generates large rotational displacement, it should be noted that accurately simulating the joint’s actual working conditions is quite challenging. Therefore, in this section, a simplified structural approach is adopted merely to verify the feasibility of the stiffness adjustment scheme and qualitatively analyze the stiffness adjustment characteristics. The phenomena obtained from the simulation data are valuable, but they cannot be used as a comparative reference for the experiment. The simulation outcomes thus serve primarily to validate the working principle and inform phenomenological understanding rather than to establish precise numerical benchmarks.

## 4. Test Device and Experiments

To bridge theoretical analysis with practical performance, this section constructs a dedicated testing apparatus for the joint, experimentally verifies its variable-stiffness capabilities under controlled conditions, and monitors load-bearing states to characterize real-world operational behavior.

### 4.1. Test Platform and Device

**Figure 16:**
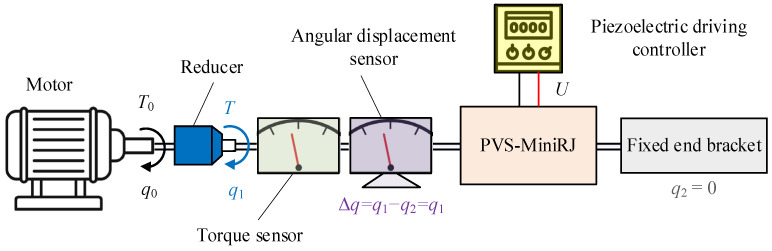
Power and movement transmission path of the test platform.

**Figure 17:**
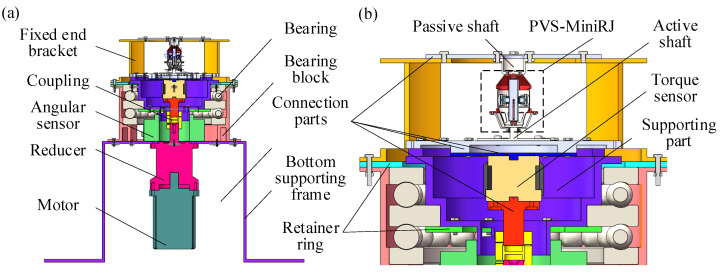
3-D model drawing of the test platform. (**a**) Overall sectional view. (**b**) Partial sectional view of the joint and sensors installation.

A test platform is constructed to verify the variable stiffness performance of the PVS-MiniRJ, as illustrated in Figure 16. The 3-D model of the test platform is shown in Figure 17. The external torque load *T*_0_ and angular displacement *q*_0_ are input to the motor and transmitted through a reducer and a coupling. Then they are converted into *T* and *q*_1_, which are delivered to the active shaft. Finally, the output is transmitted through the PVS-MiniRJ to the passive shaft. A torque sensor and angular displacement sensor are positioned between the power input and the joint. The torque sensor is connected to the active shaft for monitoring the joint torque *T*. The angular displacement sensor is mounted on a bottom supporting frame with its inner ring connected to the active shaft through a support, and it is used to monitor the angular displacement *q*_1_ of the active shaft. For the static mechanical test, the passive shaft is connected to a fixed end bracket, so *q*_2_ = 0, meaning the relative angular displacement between the active and passive shafts could be expressed as Δ*q* = *q*_1_ − *q*_2_ = *q*_1_.

**Figure 18:**
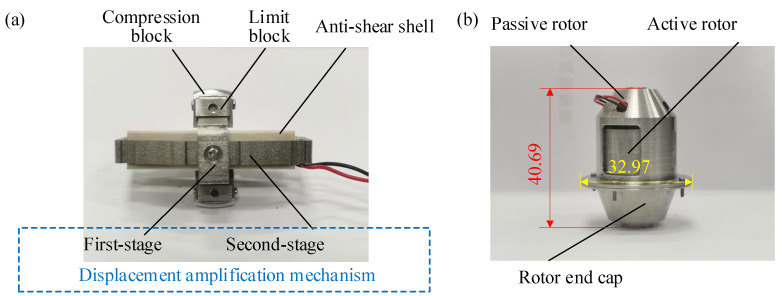
Working prototype of the PVS-MiniRJ. (**a**) Variable stiffness driving component. (**b**) Overall dimensions and appearance of the joint (unit: mm).

To facilitate testing and installation, the connection interface is appropriately modified, and then a working prototype of the PVS-MiniRJ is fabricated, as shown in Figure 18. During assembly, the components are installed sequentially from the innermost to the outermost layer. As depicted in Figure 18a, the variable stiffness driving component consists of two-stage displacement amplification mechanisms, compression blocks, and limit blocks. The overall assembly of the prototype is shown in Figure 18b. Using the caliper and the electronic balance, and taking the average of three measurements, the length, diameter, and weight of the PVS-MiniRJ are measured. As shown in Table 3, the length is 40.69 mm, the maximum diameter is 32.97 mm, and the weight is 43.43 g. The PVS-MiniRJ is equipped with several outlets for connecting the power supplies of the piezoelectric stack actuator and strain gauges.

**Figure 19:**
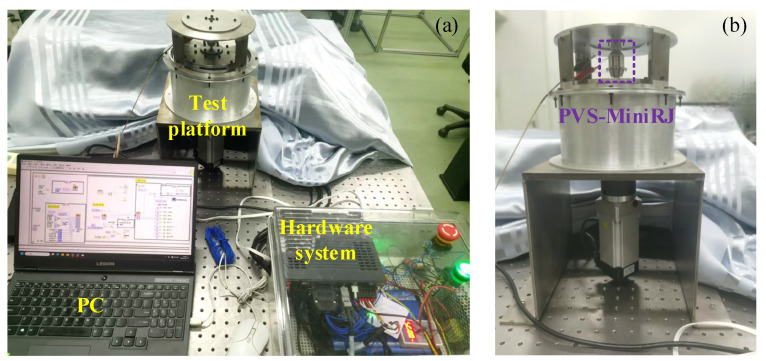
Mechanical test device for the PVS-MiniRJ. (**a**) Overall diagram of control, drive, and mechanical components. (**b**) Test platform.

The testing setup primarily consists of a PC, a hardware system, and a test rig, all mounted on an optical platform, as shown in Figure 19. The hardware system includes a power supply, motor driver, motion controller, data acquisition card, and switches. The PC is equipped with control and data acquisition software, which communicates with the controller and data acquisition card. By adjusting the input voltage *U* to the piezoelectric stack and the motor input angle Δ*q*, and meanwhile collecting data from the torque sensor and angular displacement sensor, the variable stiffness characteristics of the joint are calculated and analyzed.

### 4.2. Stiffness Characteristic Test

There are two main methods for measuring the stiffness characteristics of the joint. The first method involves applying a known torque *T* by the motor to the active shaft of the joint, measuring the angular displacement Δ*q* of the active shaft, and calculating the joint stiffness *K*. The second method involves applying a known angular displacement Δ*q* by the motor to the active shaft and measuring the resulting torque *T* to characterize the stiffness *K*. This study employs the second method and conducts multiple tests by adjusting the driving voltage *U* applied to the piezoelectric stack in order to verify its variable stiffness performance. To ensure that the verification is convincing, two sets of displacement amplification mechanisms with different dimensional parameters are configured.

(1) Test 1

**Figure 20:**
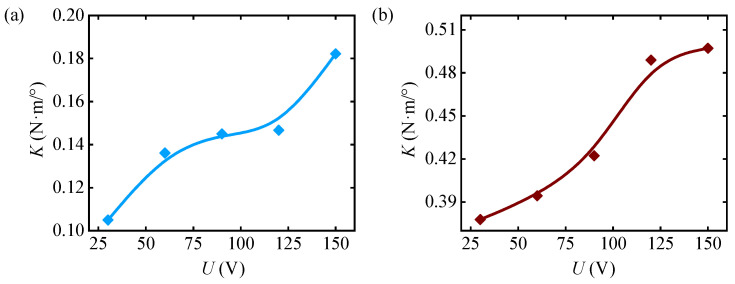
Relationship curves between driving voltage *U* of the piezoelectric stack and joint stiffness *K*. (**a**) Test 1. (**b**) Test 2.

In the first-stage displacement amplification mechanism, the thicknesses of the beam-type hinges are 0.3 mm. In the second-stage displacement amplification mechanism, the thicknesses of the beam-type hinges are 0.25 mm. The motor input angle is 1.8°. The torque data measured with varying supply voltages to the piezoelectric stack are used to calculate the joint stiffness values, as shown in Table 4. The relationship between stiffness and voltage is plotted in Figure 20a. The measured data indicate that as the supply voltage to the piezoelectric stack increases, the joint stiffness also increases. When the voltage *U* increases from 30 V to 150 V, the joint stiffness *K* increases from 0.1050 N·m/° to 0.1822 N·m/°.

(2) Test 2

In the first-stage displacement amplification mechanism, the thicknesses of the beam-type hinges are 0.4 mm. In the second-stage displacement amplification mechanism, the thicknesses of the beam-type hinges are 0.3 mm, with the other parameters remaining unchanged. Under this configuration, thicker beams in the displacement amplification mechanism have larger stiffness, leading to them being more susceptible to damage around weak structures. Therefore, to prevent overloading due to the joint rotation, the motor input angle is reduced to 0.36°. Similarly, the relationship between stiffness and voltage is plotted in Figure 20b. The measured data indicate that as the supply voltage *U* increases from 30 V to 150 V, the joint stiffness *K* increases from 0.3778 N·m/° to 0.4972 N·m/°.

Both tests conclude that as the voltage increases, the stiffness gradually increases, thereby demonstrating that the joint has the ability to adjust stiffness. This further confirms the feasibility of using a piezoelectric stack as a driving element, leveraging the inverse piezoelectric effect to adjust stiffness via expansion and contraction. In the configuration of Test 2, the joint stiffness is greater than in Test 1, mainly due to the deformation capability of the displacement amplification mechanism. In Test 1, the relatively low stiffness of the flexible hinge limits the ability of the displacement amplification mechanism to exert force outward because of the resistance of the active rotor to its own deformation. Thus, the interaction between the active and passive rotors is weaker, resulting in lower joint stiffness. Conversely, Test 2 features a flexible hinge with higher stiffness, enhancing the displacement amplification mechanism’s ability to exert force outward. This results in a more pronounced interaction between the active and passive rotors, and consequently, higher joint stiffness.

### 4.3. Mechanical Monitoring Test

As analyzed in Section 3.3, the joint stiffness is influenced by the driving force *F*_out_ and the output displacement *x*_out_ from the piezoelectric drive component, and the applied torque *T*. Given that the driving force and output displacement depend on the voltage *U*, the joint stiffness *K* is determined by the voltage *U* of the piezoelectric driving assembly and the torque *T*. This relationship can be written as

(17) $$K=K\left(U,T\right)$$

**Figure 21:**
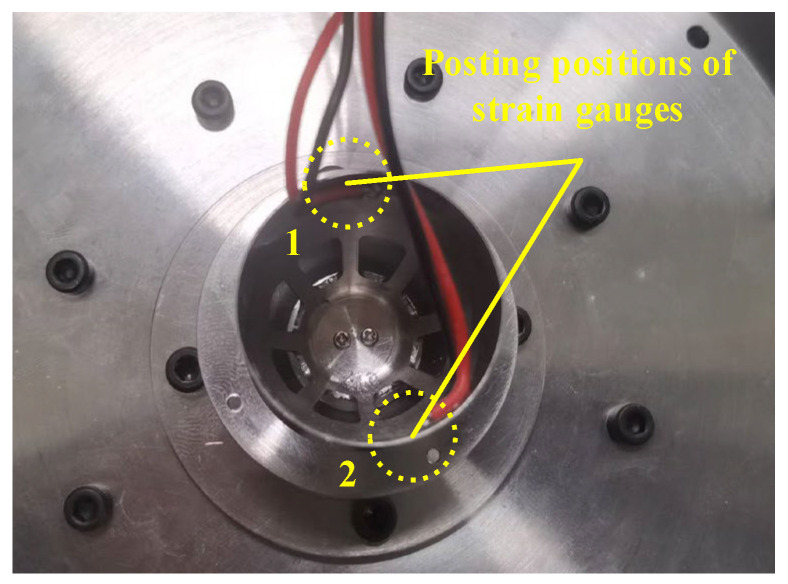
Posting positions of strain gauges installed inside the active rotor.

Therefore, to adjust the stiffness of the joint controllably, it is necessary to monitor the torque *T* in real time while adjusting the voltage *U* according to the force conditions. As described in Section 2.3.5, two strain gauges are attached to the inner surface of the flexible cylindrical section of the active rotor, as shown in Figure 21. The strain gauges are powered by a 5 V supply, with the positive and negative terminals connected to a quarter-bridge circuit.

**Figure 22:**
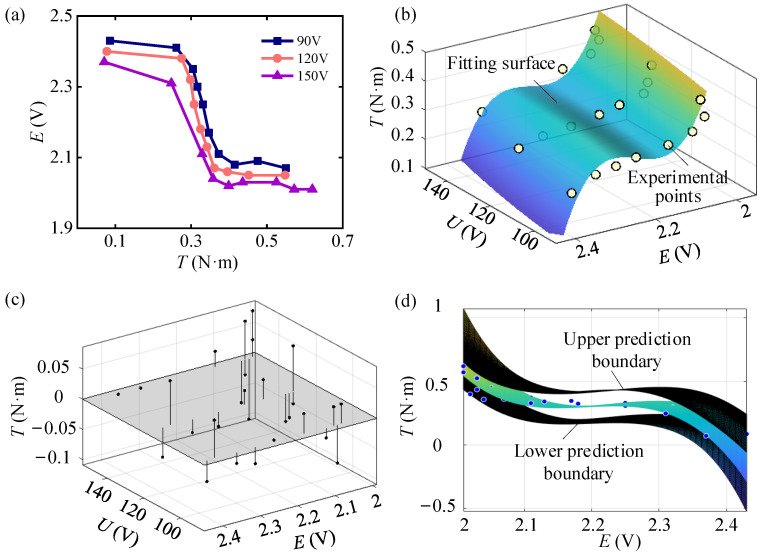
Mechanical response under load during the test. (**a**) *T*-*E* relationship curves under different values of *U*. (**b**) Comparison between the surface fitted by the least squares algorithm and the measured data. (**c**) Residual plots. (**d**) Prediction boundary of 90% confidence interval.

During the actual testing, a specific functional relationship was observed between the torque *T*, strain voltage *E*, and voltage *U* of the piezoelectric stack. Initially, the driving voltage value *U* of the piezoelectric stack was fixed and the changing torque *T* was applied through the motor. In the meantime, the corresponding strain voltage *E* was measured, and then the test was repeated with different values of *U*. During the measurements, it was observed that the voltage of strain gauge 2 remained constant. The possible reasons for this are that strain gauge 2 detached due to poor adhesion, or it was damaged during movement, which rendered it nonfunctional. Analyzing the voltage data of strain gauge 1, the corresponding relationship between *T*, *E*, and *U* is obtained, as shown in Figure 22a.

According to Figure 22a, the relationship between *T*, *E*, and *U* exhibits clear nonlinear characteristics. Using the least squares method for fitting, the fitting equation is defined as

(18) $$T=a^{T}\varphi =\sum_{i=1}^{n}a_{i}\varphi_{i}$$

where the basis functions *φ_i_* (*i* = 0, 1, …, *n*) are linearly independent orthogonal functions, the number of basis functions is *n* + 1, and *a_i_* represents the coefficients to be determined. The maximum degree of the fitting equation is 3, where the maximum degree of *U* is 2 and *E* is 3. Therefore, the expression for the basis function vector can be defined as

(19) $$\varphi =\left[\begin{array}{ccccccccc} 1 & U & E & U^{2} & UE & E^{2} & U^{2}E & UE^{2} & E^{3} \end{array}\right]$$

So *n* = 8. The normal equation is

(20) Ga=d

where

(21) $$a={\left[\begin{array}{ccccc} a_{0} & a_{1} & a_{2} & \cdots & a_{8} \end{array}\right]}^{T}$$

(22) $$d={\left[\begin{array}{ccccc} d_{0} & d_{1} & d_{2} & \cdots & d_{8} \end{array}\right]}^{T}$$

(23) $$G=\left[\begin{array}{cccc} \left(\varphi_{0},\varphi_{0}\right) & \left(\varphi_{0},\varphi_{1}\right) & \cdots & \left(\varphi_{0},\varphi_{8}\right)\\ \left(\varphi_{1},\varphi_{0}\right) & \left(\varphi_{1},\varphi_{1}\right) & \cdots & \left(\varphi_{1},\varphi_{8}\right)\\ \cdots & \cdots & \cdots & \cdots \\ \left(\varphi_{8},\varphi_{0}\right) & \left(\varphi_{8},\varphi_{1}\right) & \cdots & \left(\varphi_{8},\varphi_{8}\right) \end{array}\right]$$

where the inner product of the basis functions is $\left(\varphi_{i},\varphi_{j}\right)=\sum_{k=0}^{m}\varphi_{i}\left(U_{k},E_{k}\right)\varphi_{j}\left(U_{k},E_{k}\right)$, the inner product of the orthogonal functions and the measured data is $d_{i}=\left(\varphi_{i},T\right)=\sum_{k=0}^{m}\varphi_{i}\left(U_{k},E_{k}\right)T_{k}$. *m* + 1 is the number of data sets, where *m* = 28. Therefore, the value of the undetermined coefficient vector ***a*** can be solved, and the expression of the fitting function is obtained as

(24) $$\begin{array}{c} T=617.2-0.8045U-791.5E+0.0006065U^{2}+0.6636UE\\ +338.9E^{2}-0.0002781U^{2}E-0.1352UE^{2}-48.44E^{3} \end{array}$$

The comparison between the fitted function and the measured data is shown in Figure 22b, and the prediction residual data of *T* is shown in Figure 22c. By calculating the goodness of fit for the surface, *R*^2^ = 0.8828, SSE = 0.0618, and RSME = 0.05559, which demonstrates that Expression (24) has a high fitting accuracy. In addition, taking a 90% confidence interval, the uncertainty range of the coefficient is obtained, and the upper prediction boundary and the lower prediction boundary of the surface are determined, as shown in Figure 22d. This shows the possible range of the fitting surface. It can be seen that most of the data fall within the predicted envelope range, which verifies the reliability of the fitting function in the working condition range.

### 4.4. Error Analysis

The error sources in the test include the gap error between the rotors, the machining consistency of the rotating components, friction loss, and so on. The uncertainty of the gap between the active rotor and the passive rotor introduces nonlinear characteristics to the PVS-MiniRJ. Although human intervention in the assembly process can minimize these effects, it is still difficult to ensure that the assembly quality and test results are completely controllable. Rotors are thin cylindrical shell structures, and the consistency of their thickness and radius also affects the stability of the joint force and motion transmission and stiffness adjustment process. In addition, frequent friction and extrusion between the active rotor and the passive rotor inevitably cause material loss, which affects the consistency of the stiffness and force test data each time.

Although such errors caused by manufacturing and machining processes have adversely affected the function of the PVS-MiniRJ, the novel variable stiffness scheme and integrated joint design method proposed in this paper provide a new concept and technical path for the design of variable stiffness joints.

## 5. Conclusions

This paper presents the PVS-MiniRJ, a piezoelectric-driven miniature rotary joint (40.69 mm length, 32.97 mm diameter, 43.43 g weight) with integrated variable stiffness and torque sensing, designed for lightweight deformable mechanisms. Its key contributions are summarized as follows:

(1) Novel stiffness adjustment principle. This paper proposes a new method for joint stiffness control, exploiting piezoelectric stack expansion/contraction to dynamically regulate compressive and frictional forces between active and passive rotors. This enables direct actuation, rapid response, and a compact design. A beam-type flexure hinge displacement amplifier compensates for limited piezoelectric stroke. Integrated guiding/anti-shear components ensure reliability, while embedded strain gauges provide miniaturized sensing, achieving seamless actuation–sensing integration within the joint.
(2) Stiffness-oriented mechanical modeling and simulation. This paper develops a detailed mechanical model linking the piezoelectric stack voltage, which is the control input, to active rotor deformation, which is the monitored variable. Comparative analysis reveals that the beam-type flexure hinge offers smoother deformation and superior force distribution versus circular hinges. Finite element simulations validate the stiffness adjustment concept and elucidate the influence of the piezoelectric force *F*_out_, displacement *x*_out_, and external torque *T* on stiffness modulation.
(3) Experimental validation and mechanical monitoring. These demonstrate significant variable stiffness capability through static load testing: the joint stiffness *K* ranges from 0.105 to 0.1822 N·m/° and 0.3778 to 0.4972 N·m/° for different configurations at *U* = 90 V to 150 V. The sensor calibration establishes relationships between the torque *T*, strain voltage *E*, and piezoelectric voltage *U*, enabling real-time mechanical state monitoring and closed-loop controllable stiffness adjustment.

## Figures and Tables

**Table 1:** Dimensions and electrical parameters of MTP150/5×5/30 square piezoelectric ceramic stack.

Length	Width	Thickness	Maximum Displacement	Stiffness	Maximum Output Force	Resonance Frequency
5 mm	5 mm	30 mm	32 μm	31.25 N/μm	1000 N	34 kHz

**Table 2:** Parameters and static mechanical simulation results of the first-stage displacement amplification mechanism.

No.	Hinge Type	Parameters			Magnitude of Resistance Force to Deformation					
					*F*_e_ = 5 N		*F*_e_ = 10 N		*F*_e_ = 20 N	
		*R* or *b*	*t*_1_ or *t*_2_	*h*_1_ or *h*_2_	*σ*_Vmax_	*λ*_1_	*σ*_Vmax_	*λ*_1_	*σ*_Vmax_	*λ*_1_
1	Circular	0.35 mm	0.4 mm	1.5 mm	115 MPa	2.6	112 MPa	2.3	99 MPa	1.7
2	Beam	2.5 mm	0.4 mm	1.5 mm	43 MPa	2.8	49 MPa	2.4	88 MPa	1.5

**Table 3:** Size and quality parameters of the PVS-MiniRJ.

Measurement Number	1	2	3	Average
Length (mm)	40.68	40.68	40.70	40.69
Maximum diameter (mm)	32.96	32.97	32.97	32.97
Weight (g)	43.42	43.43	43.44	43.43

**Table 4:** Torque, flexible rotation, and stiffness test data.

*U* (V)	Test 1			Test 2		
	Δ*q* (°)	*T* (N·m)	*K* (N·m/°)	Δ*q* (°)	*T* (N·m)	*K* (N·m/°)
30	1.8	0.189	0.1050	0.36	0.136	0.3778
60	1.8	0.245	0.1361	0.36	0.142	0.3944
90	1.8	0.261	0.1450	0.36	0.152	0.4222
120	1.8	0.264	0.1467	0.36	0.176	0.4889
150	1.8	0.328	0.1822	0.36	0.179	0.4972

## Data Availability

The original contributions presented in this study are included in the article. Further inquiries can be directed to the corresponding author.

## Abbreviations

The following abbreviations are used in this manuscript:

PVS-MiniRJ	Piezoelectric-Actuated Variable Stiffness Miniature Rotary Joint
